# The Relationship Between Hand Abnormalities and Diabetic Retinopathy in Patients with Type 2 Diabetes Mellitus

**DOI:** 10.3390/jcm13226723

**Published:** 2024-11-08

**Authors:** Feyzahan Uzun, Hüseyin Fındık, Muhammet Kaim, Murat Yıldırım

**Affiliations:** 1 Department of Ophthalmology, School of Medicine, Recep Tayyip Erdogan University, 53100 Rize, Turkey; huseyin.findik@erdogan.edu.tr (H.F.); muhammet.kaim@erdogan.edu.tr (M.K.); 2 Department of Physical Medicine and Rehabilitation, School of Medicine, Recep Tayyip Erdogan University, 53100 Rize, Turkey; murat.yildirim@erdogan.edu.tr

**Keywords:** diabetic retinopathy, polyneuropathy, Quick DASH score, type 2 diabetes mellitus

## Abstract

**Background:** The link between diabetes mellitus (DM) and pathological conditions of the hand has been previously investigated. Retinopathy is one of the most common microvascular complications of DM. In this study, we aimed to evaluate the relationship between hand abnormalities and retinopathy in patients with type 2 DM. **Methods:** Patients with type 2 DM were assessed for hand abnormalities using tactile, functional, and sensory tests. The patients were evaluated electrodiagnostically for carpal tunnel syndrome and polyneuropathy (PNP). A comprehensive ophthalmologic examination was performed to diagnose diabetic retinopathy (DR). Subjective disability and quality of life were evaluated using the Quick DASH questionnaire. The duration of the disease and HbA1c levels were noted. **Results:** A total of 60 patients with type 2 DM (32 female, 28 male) were recruited for this study. The mean age was 55.1 ± 5.6 years. Among the 60 diabetic patients examined, 15 (25%) were diagnosed with DR. The mean duration of DM was 86.86 ± 51.69 months in patients without DR and 144.75 ± 82.96 months in patients with DR. The mean HbA1c level was 8.86% in the DR group and 8.64% in the non-DR group. PNP was the only hand abnormality that showed a significant association with retinopathy (*p* = 0.011). **Conclusions:** PNP might be used as a clue to the presence of retinopathy, especially in long-lasting diabetes. Particular attention should be given to hand abnormalities, especially in patients with PNP, due to their association with DR.

## 1. Introduction

Diabetes mellitus (DM) is a major public health issue known for its serious microvascular and macrovascular complications. These complications are thought to stem from increased aldose reductase activity and activation of the polyol pathway [1], leading to significant morbidity and mortality and a substantial decline in quality of life (QoL) [2]. In addition to causing microvascular complications, such as neuropathy, nephropathy, and retinopathy, DM also impacts the musculoskeletal system, including the hands [3,4]. Various hand conditions have been linked to DM, including stenosing tenosynovitis, Dupuytren’s contracture [5], carpal tunnel syndrome (CTS), limited joint mobility (LJM), pseudoscleroderma, cheiroarthropathy, and peripheral neuropathy (PNP) [4,6]. Although the precise mechanisms of these musculoskeletal problems are not fully understood, researchers suggest that they may involve increased collagen deposition, abnormal collagen cross-linking, and glycosylation [4,7].

Diabetic retinopathy (DR), the second leading cause of blindness globally, has received significant attention due to the potential for early detection and treatment [8,9]. Timely diagnosis and intervention are essential for effective management, and as a result, screening for DR is now a standard practice worldwide [10]. Both diabetic hand dysfunction and DR have been associated with longer disease duration and poor metabolic control [7,11]. Furthermore, diabetic microvascular complications, including retinopathy and neuropathy, are often linked with musculoskeletal complications and hand abnormalities [7,12,13]. However, it remains unclear whether these hand abnormalities serve as indicators of current diabetic microvascular complications or as early markers of their onset [4].

This study aims to evaluate the hand disorders associated with diabetes and explore the relationship between these abnormalities and diabetic retinopathy (DR).

## 2. Materials and Methods

In this study, a total of 60 patients with type 2 DM were included. This study was conducted in accordance with the Declaration of Helsinki and approved by the local ethics committee (IRB No: 2024/240). All patients were informed about this study, and written consent was obtained for participation. 

The exclusion criteria included ages of >65 or <30 years; any thyroid or parathyroid pathologies; serum abnormalities in vitamin D, vitamin B12, phosphorus, or calcium; cervical disc diseases; a history of cerebrovascular disease; primary rheumatological or neuromuscular diseases that could affect hand functions; and hand trauma. Demographic data and clinical information, including age, sex, duration of diabetes, glycosylated hemoglobin levels (HbA1c), and fasting and postprandial blood sugar levels, were collected. Indirect ophthalmoscopy was performed on all patients by an ophthalmologist after pupil dilation. A series of physical examinations and electrophysiological studies and a questionnaire were conducted to investigate hand abnormalities.

### 2.1. Sensorial Examinations

#### 2.1.1. Light-Touch Perception

Light-touch perception was evaluated by Semmes–Weinstein 10 g-monofilament examination (SWME), a nylon filament embedded in a plastic handle [14]. The SWME was conducted using a 5.07/10 g-monofilament applied to hand regions innervated by both median and ulnar nerves. The 5th finger, ulnar side of the 4th finger, and hypothenar region were tested for the ulnar nerve, while the 1st, 2nd, and 3rd fingers; the radial face of the 4th finger; and the thenar region were tested for the median nerve. The test was repeated three times on each region in an arrhythmic manner by an independent examiner and was considered normal when the patient correctly perceived 2 out of 3 attempts.

#### 2.1.2. Vibration Testing

Vibration testing using the on–off method was conducted with a 128 Hz tuning fork applied to the thenar and hypothenar regions as well as the 1st and 5th fingers. Each patient was asked to report their perception of the beginning and cessation of the vibration sensation upon dampening. The test was conducted twice on each region, and the responses were recorded as either present or absent [15].

#### 2.1.3. Two-Point Discrimination Test

Two pins were simultaneously applied to the phalanges to measure the patients’ ability for two-point discrimination perception, with ≤6 mm considered normal [14].

#### 2.1.4. Heat Sensation Tests

To assess heat sensation, we employed the hot–cold tube test, where the patients were exposed to temperatures of 100 °C for heat perception and 0 °C for cold perception.

### 2.2. Special Tests

#### 2.2.1. Tinel’s Sign (Median Nerve Tap Test)

This test was performed by lightly percussing over the median nerve to elicit a sensation of tingling or “pins and needles” in the distribution of the nerve, particularly in the 1st finger and thenar region [16]. 

#### 2.2.2. Phalen’s Test (Wrist Flexion Test)

The patients were asked to hold their wrists in complete and forced flexion, pressing the dorsal surfaces of both hands together for 30–60 s. They were then assessed for the characteristic symptoms of median nerve compression, such as a burning, tingling, or numb sensation over the 1st to 4th fingers and the thenar region [16].

#### 2.2.3. Reverse Phalen’s Test (Wrist Extension Test)

This test is performed by instructing a patient to maintain full wrist and finger extension for two minutes. The patient is then assessed for the presence of burning, tingling, or numb sensations in the 1st finger and thenar region [16].

The presence of symptoms in any of the Tinel, Phalen, or reverse Phalen tests in at least one hand was considered positive, while the absence of symptoms in both hands was recorded as negative. 

#### 2.2.4. Prayer Sign (Palm Press Test)

The patients were instructed to position their hands opposite to one another vertically, with elbows flexed and wrists extended. A positive prayer sign, indicative of LJM, was identified by the patient’s inability to fully approximate the palmar surfaces of the fingers. The severity of this condition was categorized as follows: Grade 0, normal findings; Grade 1, mild flexion deformity that can be corrected with passive manipulation; and Grade 2, severely fixed and irreversible flexion deformity [17].

#### 2.2.5. Quality of Life (QoL)

Abilities in daily activities and QoL were assessed using the Quick DASH (Disability of the Arm, Shoulder and Hand) scoring system. The Quick DASH questionnaire is scored in two components: the disability/symptom section (11 items, scored 1–5) and the optional high-performance sport/music or work modules (4 items, scored 1–5). However, we excluded the optional part, and the scores were calculated as previously defined, with a score of 0 representing minimum disability and a score of 100 representing maximum disability [18] (Table 1).

#### 2.2.6. Electrophysiological Studies

A Nihon Kohden Neuropack 2000 (Nihon Kohden Corporation, Tokyo, Japan), a 2-channel electroneuromyography (ENMG) system, was used by an experienced electromyographer who was blinded to the physical examination records. The CTS and PNP protocols were studied according to the guidelines of the American Association of Electrodiagnostic Medicine. CTS was graded as none, mild, moderate, or severe [19], and PNP was evaluated as either present or absent.

### 2.3. Statistical Analysis

The data were tested for normal distribution using the Shapiro–Wilk test. Normally distributed categorical variables were summarized as percentages, and descriptive statistics for the Quick DASH score were reported as interquartile ranges. Adjustment for association with retinopathy was performed using the chi-square Fisher exact test and the Mann–Whitney U test (for the Quick DASH scores). Spearman’s correlation coefficient was used to compare individual groups. Statistical significance was defined as *p* < 0.05. SPSS statistical software (SPSS 15.0 for Windows; SPSS, Chicago, IL, USA) was used for all statistical calculations. An a priori power analysis was conducted using G*Power version 3.1.9.7 to determine the minimum sample size required to test the study hypothesis. The results indicated that a total sample size of a minimum of N = 44 would be required to achieve 80% power for detecting a large effect (0.5), with a significance criterion of α = 0.05, for a chi-square test. 

## 3. Results

A total of 60 patients with type 2 DM, with a mean age of 52.1 ± 5.6 years (range: 37–66), were included in this study. The female subjects slightly outnumbered the males, comprising (53.3%, *n* = 32) compared with 46.6% (*n* = 28). The mean duration of DM was 86.86 ± 51.69 months in patients without DR and 144.75 ± 82.96 months in patients with DR. The mean HbA1c level was 8.86% in the DR group and 8.64% in the non-DR group. All subjects were right-handed, and hand examinations were conducted using the results from their right hands. 

Of the 60 diabetic patients evaluated, 15 (25%) were diagnosed with DR, while the remaining 45 (75%) did not exhibit any signs of retinopathy. Of the 15 patients with retinopathy, only 3 were diagnosed with proliferative retinopathy. Table 2 presents the duration of the disease and the HbA1c levels stratified by the presence or absence of retinopathy. The mean HbA1c level (8.86%) was higher in patients with DR; however, this difference did not reach statistical significance when compared to patients without retinopathy (8.64%) (*p* = 0.715). 

### 3.1. Sensorial Examinations vs. Retinopathy

Light-touch perception in the SWME at the ulnar nerve region was diminished in 16 patients (26.6%) and normal in 44 patients (73.4%). Retinopathy was diagnosed in 4 patients (25%) with diminished perception and in 11 patients (25%) with normal perception. No significant association was found between the ulnar SWME testing and retinopathy (*p* = 0.753). Similarly, in the median nerve region, 15 patients (25%) had diminished light-touch perception, while 45 patients (75%) had normal perception. Retinopathy was diagnosed in 5 patients (33.3%) with diminished perception and in 10 patients (22.2%) with normal perception. Again, no significant association was observed between the median SWME results and retinopathy (*p* = 0.326) (Table 3). All patients had intact vibration sensation and two-point discrimination. One patient with impaired heat sensation and four patients with impaired cold sensation did not have retinopathy. 

### 3.2. Tinel’s Sign (Median Nerve Tap Test), Phalen’s (Wrist Flexion Test) and Reverse Phalen’s (Wrist Extension Test) Tests, and Prayer Sign (Palm Press Test) vs. Retinopathy

Among those with a positive Tinel sign, 5 patients (23.8%) had retinopathy and 16 patients (76.2%) did not. Neither Tinel’s sign, Phalen’s test, nor the reverse Phalen test showed any significant association with retinopathy (*p* = 0.741, *p* = 1.00, and *p* = 0.744, respectively). Patients were also graded for the prayer sign, which is associated with limited joint mobility, as follows: grade 0 (33.3%, *n* = 20), grade 1 (51.6%, *n* = 31), and grade 2 (15.1%, *n* = 9). The prayer sign likewise showed no significant association with retinopathy (Table 3). 

### 3.3. Quick DASH Scores vs. Retinopathy

The Quick DASH scores were not found to be significantly correlated with the presence of retinopathy (z = 0.038, *p* = 0.970). The median Quick DASH scores were 23.13 (IQR = 33.4) for patients with retinopathy and 16.91 (IQR = 38.7) for those without (Table 4).

### 3.4. Electrodiagnostic Tests vs. Retinopathy 

CTS was not found to be significantly associated with retinopathy. The relationship between CTS severity and retinopathy is shown in Table 3. Of the 10 patients with PNP, 6 (60%) had retinopathy. PNP was identified as significantly associated with the presence of DR (*p* = 0.011) (Table 3).

## 4. Discussion

In this study, we evaluated hand disorders associated with DM and investigated the relationship between hand abnormalities and DR. Our findings confirmed a consistent association between peripheral neuropathy in the hands and DR in individuals with type 2 DM.

The manifestations of DM in the hand have been widely discussed over the past three decades, though the exact prevalence remains unclear. DM has been associated with several hand conditions, including stenosing tenosynovitis (trigger finger), Dupuytren’s contracture, CTS, LJM, pseudoscleroderma, and PNP [20]. Of these, LJM is one of the most extensively studied conditions under the term “diabetic hand” [21]. LJM is characterized by stiffness in the hands and an inability to fully extend the small joints, a condition clinically known as the “prayer sign” [22]. Hand stiffness as a complication of DM was first described by Lundbaek [23]. Later, Rosenbloom et al. [24] recognized an association between LJM and juvenile type 1 DM. Reports on LJM prevalence vary, ranging from 8% to 50%, likely due to differences in examination techniques and patients’ glycemic statuses [25]. LJM is notably more common in patients with DM [22,26] and is associated with longer disease duration, poor metabolic control, and microvascular complications [25,27]. Studies by Starkman et al. and Garg et al. indicate a link between LJM, retinopathy, and microalbuminuria [7,13]. Additionally, Lawson et al. reported that LJM is associated with both the presence and severity of retinopathy [12]. In our study, however, we did not find a direct association between a positive prayer sign and retinopathy. This may be due to the small sample size, the relatively short duration of diabetes among our participants, or other unaddressed confounding factors. Consistent with our findings, other studies suggest that while LJM may be linked to microvascular complications, it cannot reliably predict microvascular disease progression [28].

Tinel’s sign and Phalen’s test are two classic indicators that are highly useful in diagnosing CTS, a median nerve-entrapment neuropathy. The prevalence of DM among patients with CTS has been reported to range from 11% to 25%, while 5% to 8% of individuals with DM have CTS [29,30]. Although CTS has been linked to disease duration [29], it has not been associated with poor metabolic control or microvascular complications [31]. Studies show that the prevalence of CTS is higher in patients with type 2 DM (83.3%) than in those with type 1 DM (24.4%), and CTS has been found to correlate with both age and duration of diabetes [32]. Yamamoto et al. assessed hand function using special tests, including the prayer sign, Tinel, and Phalen tests, and electrophysiological studies in patients with DM. They found that older age, higher BMI, and longer diabetes duration were associated with hand dysfunction [33]. In a study by Pandey et al., retinopathy was present in 44.5% (89/200) of DM patients, with LJM and CTS associated with its presence [20]. In our study, we did not observe a correlation between Tinel’s sign, Phalen’s test, and retinopathy. This may be due to the relatively small sample size, the short mean duration of the disease among the participants, or other unknown confounding factors.

Diagnosing PNP, one of the most common complications of DM, is challenging because it affects different types and numbers of nerve fibers. The SWME and the 128 Hz tuning fork tests are recommended as screening tools [34]. In this study, in addition to these tests, we used two-point discrimination and thermal sensation as sensory testing tools. In patients with type 1 diabetes, thermal sensation abnormalities were demonstrated, whereas vibration sensation was not [15]. Cederlund et al. found that vibrotactile sense was symmetrically impaired in the index and little fingers in patients with DM. However, no differences in thermal sensation were observed between groups [35]. Tactile sensation was also found to be deteriorated in diabetic patients, with this impairment being primarily associated with poor metabolic control [36]. Chao et al. reported that small-fiber neuropathy, characterized by impaired thermal sensations, was identified as the most common sensory deficit in diabetes, and HbA1c levels were significantly associated with elevated thermal thresholds [37]. In diabetic PNP, temperature sensitivity is generally impaired before tactile sensation due to the vulnerability of small C-fibers and Aδ-fibers to metabolic damage. Tactile sensation, mediated by larger Aβ-fibers, tends to be affected later. While both deficits are linked to diabetic complications like retinopathy through shared mechanisms such as microvascular damage, there is no definitive evidence showing that temperature or tactile loss is more strongly associated with retinopathy [38]. The risk of developing and progressing PNP has been associated with poor glycemic control and the duration of diabetes [39]. Additionally, the severity of PNP correlates with other microvascular complications of diabetes and HbA1c levels [40]. Although PNP primarily affects the feet, it can also involve the hands; when this occurs, the disease is typically already advanced in the feet and legs. In mild cases, PNP may primarily affect small fibers, leading to a loss of temperature discrimination. In more severe cases, vibration perception, as assessed by tuning fork examination, is diminished, and abnormalities in electrophysiological studies may also become apparent [41]. Electrophysiological nerve studies, particularly electrodiagnostic tests (ENMGs), play a crucial role in diagnosing diabetic neuropathy by assessing nerve conduction velocity and identifying abnormalities in sensory and motor nerves [42]. Patients with diabetic neuropathy often exhibit an increased risk of retinopathy, reflecting shared underlying pathophysiological mechanisms such as chronic hyperglycemia and microvascular damage [42]. Consistent with our findings, we observed that PNP was directly in relation with the presence of retinopathy. Among the 15 patients with retinopathy, 5 (33.3%) also had PNP. Conversely, among the 10 patients with PNP, 6 (60%) had retinopathy. Therefore, the presence of PNP suggests a stronger association with retinopathy than the reverse. Similarly, another study found that severe retinopathy was associated with diabetic neuropathy and suggested that PNP might be a predictor marker for DR [43].

The Quick DASH questionnaire is widely used in clinical research to assess physical function and symptoms in individuals with upper-extremity musculoskeletal disorders. Studies on its reliability indicate that the Quick DASH questionnaire can be used as an alternative to the DASH questionnaire, offering similar precision in measuring disability and symptom severity across various upper limb disorders [44]. DM also impacts QoL by affecting hand performance in daily activities. A meta-analysis revealed reduced hand function, specifically in grip and pinch strength, as well as decreased QoL in patients with type 2 DM [45]. Individuals with long-term DM (>15 years) have been found to experience greater impairments in sensibility and activities of daily living [35]. In our study, we found that Quick DASH scores directly correlated with HbA1c levels but not with retinopathy.

Our study has several limitations. Non-diabetic patients could have been evaluated for the same clinical signs and tests applied to the diabetic patients, serving as a control group. Additionally, this study could be replicated with a larger patient cohort, including individuals with a longer duration of disease or varying degrees of metabolic control.

In conclusion, we extensively examined hand abnormalities in diabetic patients using various clinical tools and found a significant relation between retinopathy and PNP in the hands. While a minority of patients had diminished light-touch perception in the median nerve region, all patients maintained intact vibration sensation and two-point discrimination. Additionally, the presence of impaired heat or cold sensation did not correspond with the presence of retinopathy. PNP may serve as a predictor of DR, highlighting the need for more intensive ophthalmologic monitoring. Patients diagnosed with PNP should be promptly referred to ophthalmologists to prevent further retinal damage from DR, ensuring timely intervention and appropriate care. Future studies with larger populations and patients with long-standing diabetes are needed to clarify these associations.

## Figures and Tables

**Table 1:** Quick DASH scoring system questionnaire.

Quick DASH					
Please rate your ability to do the following activities in the last week by circling the number below the appropriate response.					
	No difficulty	Mild difficulty	Moderate difficulty	Severe difficulty	Unable
1-Open a tight or new jar.	1	2	3	4	5
2-Do heavy household chores (e.g., wash walls, floors).	1	2	3	4	5
3-Carry a shopping bag or briefcase.	1	2	3	4	5
4-Wash your back.	1	2	3	4	5
5-Use a knife to cut food.	1	2	3	4	5
6-Recreational activities in which you take some force or impact through your arm, shoulder, or hand (e.g., golf, hammering, tennis, etc.).	1	2	3	4	5
	Not at all	Slightly	Moderately	Quite a bit	Extremely
7-During the past week, to what extent has your arm, shoulder or hand problem interfered with your normal social activities with family, friends, neighbors, or groups?	1	2	3	4	5
	Not limited at all	Slightly limited	Moderately limited	Very limited	Unable
8-During the past week, were you limited in your work or other regular daily activities as a result of your arm, shoulder, or hand problem?	1	2	3	4	5
Please rate the severity of the following symptoms in the last week (circle number).	None	Mild	Moderate	Severe	Extreme
9-Arm, shoulder, or hand pain.	1	2	3	4	5
10-Tingling (pins and needles) in your arm, shoulder, or hand.	1	2	3	4	5
	No difficulty	Mild difficulty	Moderate difficulty	Severe difficulty	So much difficulty that I can’t sleep
11-During the past week, how much difficulty have you had sleeping because of the pain in your arm, shoulder, or hand (circle number)?	1	2	3	4	5

**Table 2:** Disease duration and HbA1C levels of the patients with and without retinopathy.

	Diabetic Retinopathy										
	Absent					Present					
	Mean	SD	Min	Median	Max	Mean	SD	Min	Median	Max	*p* Value
HbA1C	8.64	2.1	5.53	8.41	14.72	8.86	1.94	6.5	8.68	13.3	0.715
Duration of DM (months)	86.86	51.69	15	60	264	144.75	82.96	24	132	312	0.009

**Table 3:** Sensorial testing, special tests, prayer sign, CTS, and PNP vs. retinopathy.

		Retinopathy		*p*
		Absent *n* (%)	Present *n* (%)	
SWME ulnar	Normal	33 (66.7)	11 (33.3)	0.753
	Diminished	12 (75)	4 (25)	
SWME median	Normal	35 (77.8)	10 (22.2)	0.326
	Diminished	10 (66.7)	5 (33.3)	
Tinel’s sign	Negative	29 (74.4)	10 (25.6)	0.741
	Positive	16 (76.2)	5(23.8)	
Phalen’s test	Negative	33 (73.4)	12 (26.6)	1.000
	Positive	12 (80)	3 (20)	
Reverse Phalen’s test	Negative	35 (74.5)	12 (25.5)	0.744
	Positive	10 (76.9)	3 (23.1)	
Prayer sign	Grade 0	15 (75)	5 (25)	
	Grade 1	21 (67.8)	10 (32.2)	
	Grade 2	9 (100.0)	0 (0.0)	
CTS	None	32 (76.1)	10 (23.9)	
	Mild	2 (66.7)	1 (33.3)	
	Moderate	8 (66.7)	4 (33.3)	
	Severe	3 (100)	0 (0.0)	
PNP	Absent	41 (80)	9 (20)	0.011
	Present	4 (40.0)	6 (60.0)	

SWME: Semmes–Weinstein 10g-monofilament examination, CTS: carpal tunnel syndrome, PNP: polyneuropathy.

**Table 4:** Quick DASH scores vs. retinopathy.

Retinopathy	Quick DASH Score Median (IQR)	Z	*p*
Present	23.13 (32.95)	0.038	0.970
Absent	16.91 (36.93)		

## Data Availability

The original contributions presented in this study are included in the article, further inquiries can be directed to the corresponding author.
